# Effects of tender point acupuncture on delayed onset muscle soreness (DOMS) – a pragmatic trial

**DOI:** 10.1186/1749-8546-3-14

**Published:** 2008-11-25

**Authors:** Kazunori Itoh, Hideki Ochi, Hiroshi Kitakoji

**Affiliations:** 1 Department of Clinical Acupuncture and Moxibustion, Meiji University of Integrative Medicine, Hiyoshi-cho, Nantan, Kyoto 629-0392, Japan

## Abstract

**Background:**

Acupuncture is used to reduce inflammation and decrease pain in delayed onset muscle soreness (DOMS). This study investigates the efficacy of acupuncture on the symptoms of DOMS.

**Methods:**

Thirty subjects were assigned randomly to there groups, namely the control, non-tender point and tender point groups. Measurement of pain with full elbow flexion was used as indices of efficacy. Measurements were taken before and after exercise, immediately after treatment and seven days after treatment.

**Results:**

Significant differences in visual analog scores for pain were found between the control group and tender point group immediately after treatment and three days after exercise (P < 0.05, Dunnetts multiple test).

**Conclusion:**

The results show that tender point acupuncture relieves muscle pain of DOMS.

## Background

Delayed onset muscle soreness (DOMS) is a common myogenic condition with main symptoms of pain, tenderness and loss of range of movement that usually occur 24 to 48 hours when unaccustomed. While DOMS is not a serious condition, it may discourage the sufferer's participation in exercise and rehabilitation. Therapy for DOMS currently includes the use of anti-inflammatory analgesics [1], massage [2], transcutaneous electrical nerve stimulation (TENS) [3], ultrasound [4] and laser [5]. DOMS may result from an inflammatory process in muscle after exercise [6-8]. While previous studies show that acupuncture may or may not reduce inflammation and decrease pain in DOMS [9,10], we believe that response to acupuncture largely depends on the choice and number of acupoints.

Recent clinical investigations on musculoskeletal pain, such as myofascial pain syndrome and fibromyalgesia, focus on tender points and/or trigger points which have already been used for diagnosis and treatment. The present study aims to investigate localized tenderness of experimentally induced DOMS and compare the characteristics of DOMS with those of myofascial pain syndrome [11,12].

## Methods

### Subjects

Approved by the Meiji University of Integrative Medicine Ethics Committee this study included healthy student volunteers from the acupuncture school of the University (n = 30, aged 18–22 years). Volunteers were informed of the experimental procedures and agreed to participate in the present study by signing a consent form.

### Screening procedure

The exclusion criteria were that the subjects did not have current injury or pain (bone fracture, bruise and/or sprain of upper arm), consumption of any form of drugs, pregnancy, hemophilia, diabetes mellitus, asthma, weight-training, intense fear of needles and participation in a similar experiment within the past year. All subjects were instructed to avoid any form of exercise during the experiment.

### Pain induction

DOMS was induced with standard methods in the non-dominant elbow flexors (biceps brachii) of the subject [13,14]. Subjects were seated behind an inclined biceps-curl bench so that they could fully flex and extend their elbows. The maximum weight lifted with one voluntary concentric contraction was determined with free weights (a loaded dumbbell) for each subject. This maximum weight was subsequently used to exhaust the elbow flexors. A researcher lifted the weight until the subject's elbow was in a position of full flexion. The subject was instructed to lower the weight as slowly as possible until the elbow reached full extension. The researcher returned the weight to the starting position (full elbow flexion) and the process was repeated for as many times as the subject could control the speed of descent of the weight. The time of exhaustion was taken as the point at which the subject could no longer control the lowering of the weight.

### Randomization procedure

A researcher screened and enrolled subjects. After the participants completed a baseline evaluation, another researcher who was not involved with data collection randomly assigned them to one of the three treatment groups using a computer-generated (SAMPSIZE V2.0, Blackwell Science Ltd, UK), blocked random allocation sequence with a block size of three.

### Experimental conditions

#### Control group

Subjects allocated to this group rested supinely on a standard treatment plinth for a period of 10 minutes.

#### Non tender point group (non-TeP group)

Subjects in this group received needling at four non-tender and non-acupuncture points located on the lateral side of the upper arm in the indentation between the biceps brachii and the brachialis. These points were typically located on the distal third of the belly of the biceps brachii approximately over the musculotendinous junction. Disposable stainless steel needles (0.18 mm × 40 mm, Seirin, Japan) were inserted straight to a depth of 1 to 2 cm and retained in place for 10 minutes.

#### Tender point group (TeP group)

Subjects in this group received needling at the points identified (by palpation) as the three most tender after exercise. These points were typically located on the distal third of the belly of biceps brachii approximately over the musculotendinous junction. Disposable stainless steel needles (0.18 mm × 40 mm, Seirin, Japan) were inserted straight to a depth of 1 to 2 cm and retained in place for 10 minutes.

#### Both non-TeP and TeP groups

Ten minutes after exercise, treatments were given to non-TeP and TeP groups simultaneously.

The acupuncture was performed by three acupuncturists with three years of acupuncture training and one or ten years of clinical experience.

### Measurement

#### Pain measurement

Intensity of muscle pain of the arm muscle at full flexion was estimated before and immediately after exercise, immediately after treatment and one, two, three and seven days after exercise. Subjects were asked to rate their current level of pain by marking a visual analog scale (VAS). A 10 cm line appeared with 'no pain' marked at one end and 'maximum pain' marked at the other. Subjects were asked to indicate their current level of pain with full elbow flexion.

#### Experimental schedule

Thirty subjects were allocated randomly to one of the three groups. In treatment groups one and two, the VAS of the exercised muscle was measured before and immediately after exercise, immediately after and one, two, three, four and seven days after treatment. No treatment was performed in the control group, but the VAS was measured on the same schedule as the other groups. This study is an observer-blinded, randomized controlled clinical trial.

#### Blinding

The measurements were performed by an independent investigator who was not informed of the treatment allocation.

### Statistical analysis

The data are reported as means ± standard deviation (means ± SD). Repeated measures analysis of variance (ANOVA) was used for the primary measure, an inter-group of VAS scores. Repeated measures ANOVA were performed in each group for intra-group differences, followed by pair wise comparisons with Sidak correction to maintain experiment error rate below 5%. Differences between the groups at each time period were analysed with the unpaired *t *test and Dunnett's test, by which each test performed was judged against a corrected significance level as α/c, where α is significance level (0.05) and c is the number of paired comparisons. SYSTAT 11 was used for the analysis of a between-group (repeated measures ANOVA) and StatView for Windows (version 5.0) was used for the analysis of between the groups (Dunnett's test). *P *< 0.05 was considered to be statistically significant.

## Results

### Subjects

Thirty subjects (13 women, 17 men; aged 18–22 years) were randomly assigned to the groups. No differences were found among the groups in terms of baseline variables and age.

**Figure 1:**
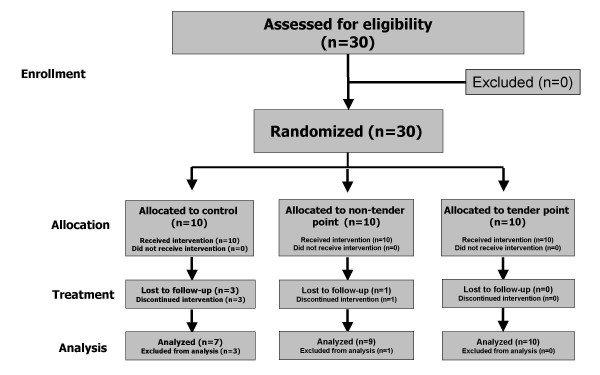
Subject participation flow in the study.

Three subjects in the control group and one subject in the non-TeP group dropped out. The reason for dropout was that they received other treatments (e.g. massage, anti-inflammatory poultice). The analyses were conducted with the data from the 26 patients who completed the study (Figure 1).

### VAS score

Immediately after exercise, subjects in all groups felt warmth and tenderness in the working muscle of the upper arm. The region of tenderness was gradually restricted to the musculotendinous junction, and a rope-like taut band was observed in the tender area after exercise on the first day of the study.

As shown in Table 1, the mean VAS scores tended to decrease from immediately after treatment, although the time courses were different for each group. There were statistically significant differences between the VAS scores of the control group and TeP group immediately (4.2 ± 2.7 vs 0.6 ± 0.9) and three days (2.4 ± 2.3 vs 0.4 ± 0.5) after treatment (*P *< 0.005 in both cases). There were no significant differences between VAS scores of the control group and non-TeP group. While the non-TeP group and TeP group reported relatively lower pain intensity than the control group, the differences were not statistically significant in ANOVA.

**Table 1:** Pain intensity scores in visual analog scale (VAS)

	**Control (n = 10)**	**Non-tender point (n = 10)**	**Tender point (n = 10)**
**Before exercise**	0	0	0
**After exercise**	4.3 (2.9)^##^	3.6 (2.6)^##^	3.8 (1.7)^##^
**Treatment**	4.2 (2.7)^##^	1.7 (2.6)	0.6 (0.9)*
**1 day**	5.4 (2.4)^##^	4.7 (2.8)^##^	3.0 (2.0)^##^
**2 days**	4.0 (2.3)^#^	3.7 (2.6)^##^	1.7 (1.9)^#^
**3 days**	2.4 (2.3)	1.2 (1.3)	0.4 (0.5)*
**7 days**	0	0	0

Data are expressed as mean (SD).

*P < 0.005: inter-group differences

^#^*P *< 0.05, ##*P *< 0.01: intra-group differences

## Discussion

A statistically significant difference was found only between the tender point acupuncture and control groups, immediately and three days after treatment. The results suggest that tender point acupuncture treatment may be effective for DOMS.

### Clinical trials

Available experimental evidence does not consistently support acupuncture as a method for pain relief [15,16]. Past studies were flawed in experimental design, sample size or control. In some randomized controlled trials of acupuncture, control groups were defined as no-treatment controls [17], pricking (without penetration) [18], minimal acupuncture (shallow and weak needling) [19] and mock TENS (without pulse) [20,21]. Moreover, negative results tended to come from studies using non-tender point acupuncture [10] and positive results from studies using non-acupuncture control groups [9]. We used no-treatment controls and non-tender point acupuncture in this study. Non-tender point acupuncture has been proposed as a sham technique [22,23], which is problematic due to the existence of diffuse noxious inhibitory controls (DNIC) phenomena. Painful stimulation inhibits pain, and DNIC has been proposed as a physiological basis of acupuncture analgesia [24,25]. In fact, six subjects complained of dull sensation (known as *deqi*) during non-tender acupuncture treatment. Therefore, we used non-tender point acupuncture as treatment group in this study.

Previous experience with acupuncture and confidence in acupuncture may influence the measurement of efficacy [24,26]. Therefore, the limitations of the present study is that the subjects were students of an acupuncture school, who had considerable knowledge of acupuncture and the special sensation of *deqi*, and who had confidence in acupuncture.

### Effects of tender point acupuncture on DOMS

Our results show that tender point acupuncture has analgesic effects. The strength of stimulation may depend on various parameters such as manipulating procedure, size of needle and site of insertion. Tender point insertion of the needle affects sensitized nociceptors, whereas non-tender point insertion does not [27,28]. Tender points are sites where nociceptors, such as polymodal-type receptors, have been sensitized by various factors [29,30]. Our data suggest that acupuncture stimulation of tender points may activate sensitized polymodal-type receptors thereby relieving pain. In clinical practice, acupuncture treatment may be effective on myofascial pain syndromes.

Barlas *et al. *concluded that acupuncture had little effect on the cardinal signs and symptoms of DOMS. We find the claim problematic. Deep pain sensation, such as muscle pain, is very complex [31]. A previous study found that the intensity of muscle pain at full flexion and full extension were different [32]. Moreover, pressure algometry used in studying muscle pain excites cutaneous receptors and may induce pain sensation in the skin [33]. In the present study, we estimated the intensity of muscle pain of the arm at full flexion.

## Conclusion

The present study shows that tender point acupuncture relieves the symptoms of DOMS. Large scale clinical trials are warranted to confirm this finding.

## Abbreviations

DOMS: delayed onset muscle soreness; TENS: transcutaneous electrical nerve stimulation; Non-TeP: non-tender point; TeP: tender point; VAS: visual analog scale; ANOVA: analysis of variance

## Competing interests

The authors declare that they have no competing interests.

## Authors' contributions

KI did the study design, acupuncture treatment and manuscript preparation. HO performed statistical analysis. HK did the study design, critical review of the manuscript and recruitment of subjects. All authors read and approved the final version of the manuscript.
